# All-suture technique for fixation of unstable displaced distal clavicle fracture

**DOI:** 10.1016/j.xrrt.2022.01.005

**Published:** 2022-02-19

**Authors:** Gregory Cunningham, L. Alejandro Culebras Almeida, Morgan Gauthier

**Affiliations:** a Division of Orthopaedics and Traumatology, University Hospitals of Geneva, Geneva, Switzerland; b Shoulder and Elbow Center, Hirslanden Clinique La Colline, Geneva, Switzerland; c Department of Orthopaedics, Balgrist University Hospital, Zürich, Switzerland

**Keywords:** Distal clavicle fracture, Osteosuture, Fixation, Cerclage

## Abstract

**Background:**

Displaced Neer type II and V clavicle fractures are usually treated surgically in active patients. However, distal fragment fixation remains a challenge, and no consensus has been established regarding the optimal surgical treatment. Osteosuture techniques have been popularized over the last decade, and multiple different techniques have been described. The aim of this study was to describe an all-suture technique in patients with displaced type II and V clavicle fractures and report its outcome in a prospective case series.

**Methods:**

Between 2017 and 2020, 15 patients with displaced acute distal clavicle fractures were treated with an all-suture open technique performed by one shoulder specialized surgeon, with a minimum follow-up of 1 year. Osteosuture repair consisted in a coracoclavicular cerclage with 4 no6 Ethibonds and a figure-of-0 and figure-of-8 fracture cerclage with 2 no2 SutureTapes. Single assessment numerical evaluation (SANE) and adjusted Constant score were recorded at 6 months and 1 year. The radiologic union was assessed on plain radiographs.

**Results:**

At 12 months, all patients reported excellent clinical results, with a mean SANE of 98.2 [± 5.2, range 80 to 100] and a mean adjusted Constant score of 99.0 [± 1.9, range 94 to 100]. One patient developed shoulder stiffness that resolved before the final follow-up. Fractures consolidated in 93% of the cases, with union happening between 3 and 6 months [range 3 to 12 months]. One patient developed an asymptomatic malunion.

**Conclusion:**

Excellent clinical and radiological outcomes can be achieved with this minimally invasive all-suture fixation technique for displaced distal clavicle fractures, which allows for an anatomic reduction and stable fixation. This pilot study showed low complications and a high level of union after a follow-up of 1 year. Among the numerous advantages are a smaller exposure than for plate fixation, avoidance of hardware-related complications such as screw failure, coracoid fracture from drilling, or rotator cuff damage caused by hook-plates. Furthermore, it avoids a reoperation to remove symptomatic hardware.

Distal clavicle fractures account for 15% of clavicle fractures.^6^ These fractures are classified into five types (according to the modified Neer classification).^32^^,^^35^ Type I fractures are extra-articular fractures occurring lateral to coracoclavicular (CC) ligaments. As the conoid and trapezoid ligaments remain intact, they are considered as stable fractures. Type II fractures are further divided into type IIA fractures that occur medially to coracoclavicular ligaments and type IIB fractures that occur between the conoid and trapezoid ligaments. Both fracture patterns show a significant medial clavicle displacement and are considered as unstable. Type III fractures are intra-articular fractures, occurring lateral to CC ligaments and extending into the acromioclavicular (AC) joint. As the conoid and trapezoid ligaments remain intact, they are considered as stable fractures. Type IV fractures are physeal fracture occurring in the skeletally immature and are considered as stable. Finally, type V are comminuted fractures, with intact conoid and trapezoid ligaments but showing a significant medial clavicle displacement and are considered as unstable.^3^^,^^33^

Neer type II and V fractures are known to be unstable with a high risk of nonunion due to the disrupted coracoclavicular ligaments. Therefore, surgical fixation remains recommended in active patients.^2^ Several fixation techniques have been reported, including locking plate, hook plate, tension band wiring, coracoclavicular fixation using a screw, a cortical button, or suture.^2^^,^^6^ The wide diversity and lack of consensus regarding treatment superiority show the challenge and complexity in managing these types of fractures.

Although locking and hook plates show good clinical outcomes, fixation of small distal fragments with locking plates may be difficult, and hook plates may cause rotator cuff lesions and requires early implant removal.^37^ Other techniques such as k-wire fixation are not recommended because of the risk of implant migration and potentially life-threatening complications.^37^

Osteosuture with or without cortical button fixation has been popularized over the last decade, and multiple different techniques have been described.^14^^,^^26^^,^^34^^,^^38^

The aim of this prospective case series was to describe a new type of all-suture technique in patients with displaced type II and V distal clavicle fractures and report its clinical and radiological outcomes.

## Materials and methods

### Patient selection

Between 2017 and 2020, all consecutive active patients, aged more than 18 years, presenting to a specialized shoulder clinic with an acute displaced Neer type II or V distal clavicle fracture and eligible for surgery were prospectively included in this study. Patients with nondisplaced fractures, significant comorbidities, or inactive were not operated on and excluded. All operations were standardized and performed by a single shoulder specialist surgeon, with a minimum follow-up of 1 year.

### Surgical technique

**Figure 1:**
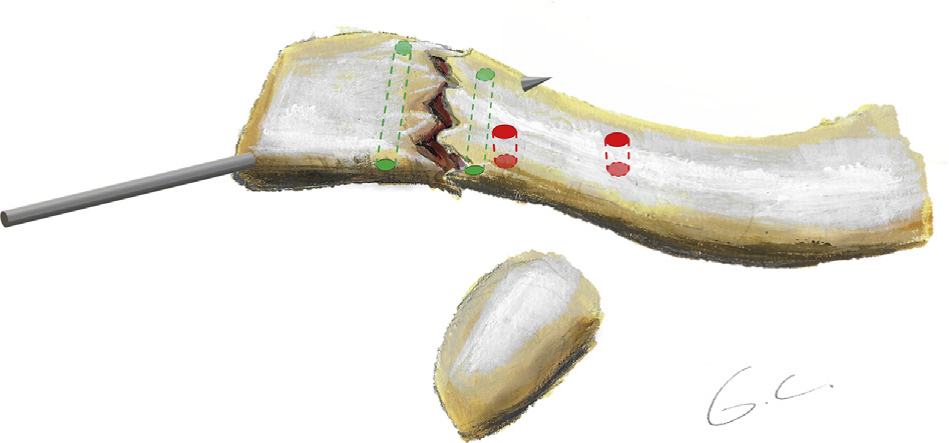
Illustration of a distal clavicle fracture reduced and fixed by a temporary k-wire. Two green lateral tunnels represent the 2 mm horizontal drilling for the fracture fixation, 2 red medial tunnels represent the 3.2 mm vertical drilling for the coracoclavicular cerclage. The most lateral is slightly anterior, and the most medial is slightly posterior to respect the anatomy of the coracoclavicular ligaments.

**Figure 2:**
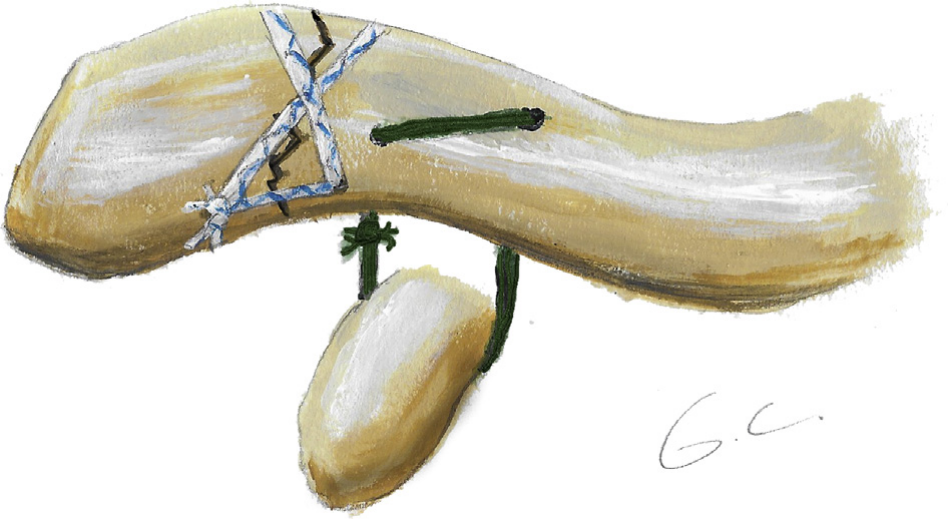
Illustration of the final construct, consisting in a coracoclavicular cerclage with 4 no6 Ethibonds and a figure-of-0 and figure-of-8 fracture cerclage with 2 no2 SutureTapes.

**Figure 3:**
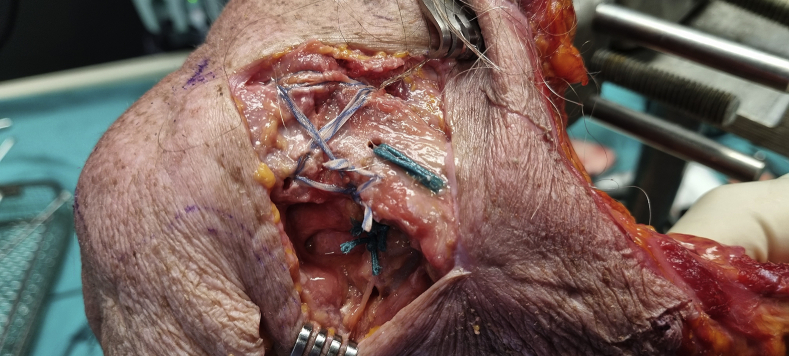
Intraoperative picture (cadaver lab) showing the final construct.

**Figure 4:**
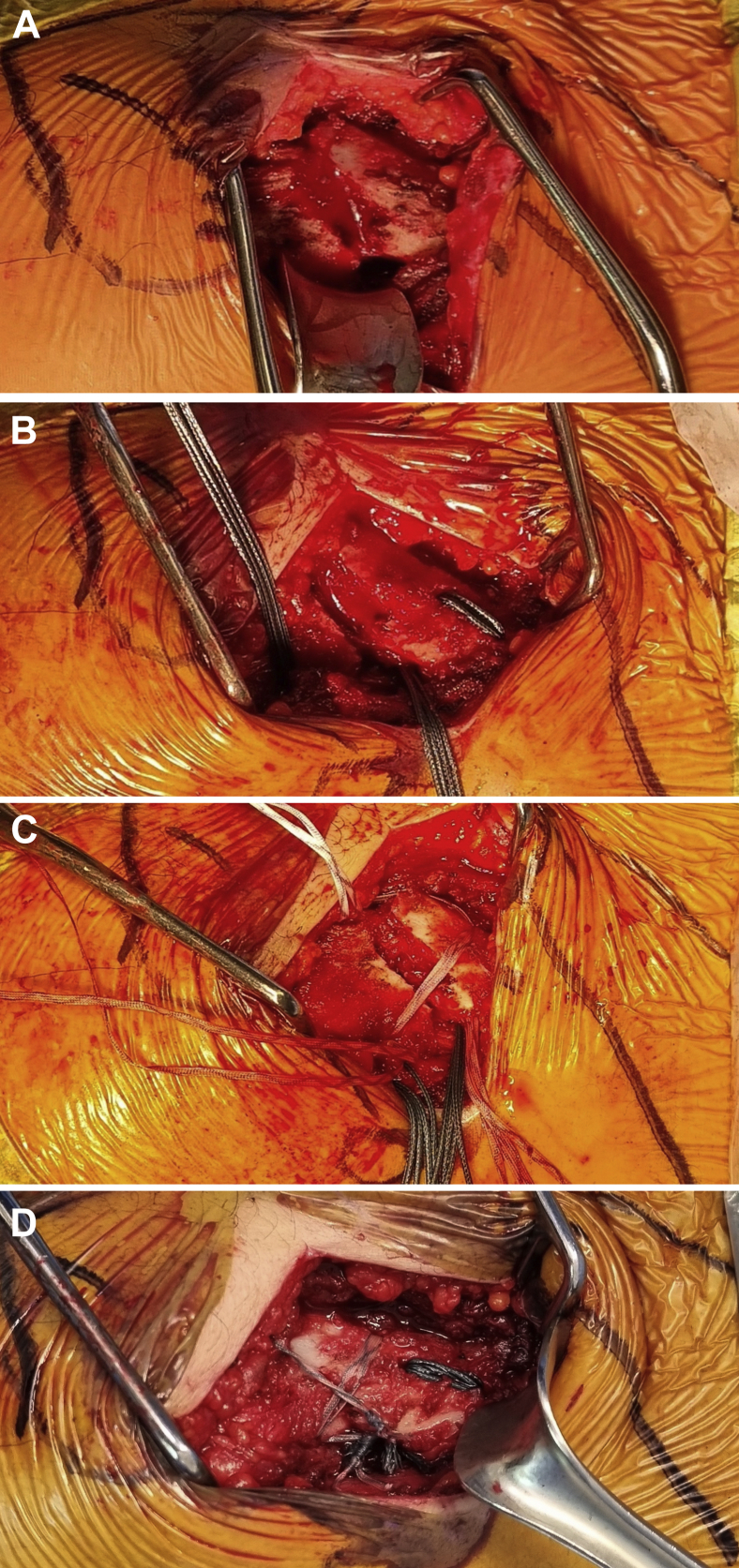
Intraoperative pictures showing the surgical steps. (**A**) The fracture is exposed through a direct 7 cm incision. (**B**) The coracoclavicular cerclage is performed using 4 no6 Ethibond sutures, which were passed under the coracoid and then passed through two 3.2 mm vertical holes in the clavicle. (**C**) Osteosuture of the fracture site using 2 no2 SutureTapes to form a figure-of-8 and a figure-of-0, passed through two 2 mm anteroposterior holes on either side of the fracture site. (**D**) The knots of the SutureTapes are tied first, and then the knots of coracoclavicular cerclage Ethibonds, using an arthroscopic knot pusher to firmly secure the knots under the clavicle.

This technique consists of a coracoclavicular cerclage and a figure-of-0 and figure-of-8 fracture cerclage (Figure 1, Figure 2, Figure 3). All surgeries were performed in a beach chair position with the patient under general anesthesia or sedation with an interscalene block. A 5-7 cm oblique incision was made from the posterolateral distal clavicle toward the coracoid process. The deltoid insertion was partially detached, and the fracture exposed (Fig. 4*A*); the tear of the coracoclavicular ligaments was confirmed in all cases. The coracoid process was exposed laterally and medially by blunt dissection through the coracoacromial ligament and the pectoralis minor, respectively. The fracture was reduced and fixed with a temporary k-wire or c-clamp (Fig. 1). The first step consisted of a coracoclavicular cerclage, using a similar technique to the one used in the same institution for acromioclavicular joint dislocation^24^: Four no6 Ethibond (Ethicon, Somerville, NJ) sutures were passed under the coracoid while protecting the neurovascular structures with a blunt Hohman retractor. Two 3.2 mm holes were drilled vertically in the clavicle, medial to the fracture, respecting the insertion site of the coracoclavicular ligaments.^5^ The 4 Ethibond sutures were passed first through the medial hole superiorly and then through the lateral hole inferiorly so that the knots could be later tied under the clavicle to avoid subcutaneous tissue irritation (Fig. 4*B*). The second step consisted of osteosuture of the fracture site (Fig. 1): Two anteroposterior 2 mm holes were drilled in the clavicle on either side of the fracture site. Two no2 SutureTapes (Arthrex, Naples, FL, USA) were passed through the drill holes to form a figure-of-8 and a figure-of-0 (Fig. 4*C*). The knots of the SutureTapes were tied first, and then the knots of coracoclavicular cerclage Ethibonds, using an arthroscopic knot pusher to firmly secure the knots under the clavicle (Fig. 4*D*). The fracture was reduced first with the help of a downward pusher to allow proper restoration of the medial fragment and avoid over reduction. Then, the coracoclavicular cerclage was fixed, and the reduction was verified visually with an image intensifier. The superior trapezodeltopectoral fascia was reconstructed using no2 Vicryl sutures (Ethicon, Somerville, NJ, USA), the wound was closed and dressed according to standard care.

### Postoperative care

Postoperatively, patients were immobilized in a sling for 6 weeks. Active wrist and elbow motion was allowed, as well as progressive passive shoulder range of motion in the plane of the scapula. Pendulum exercises were prohibited to avoid excessive tension on the repair construct. After 6 weeks, active range of motion was allowed with progressive strengthening and full return to sports activities at 3 months.

### Postoperative assessment

Patients were evaluated with physical examination and radiologic studies at 6 weeks, 12, weeks, 6 months, and 12 months. Range of motion, pain on palpation of the fracture site, visual analog scale (VAS),^11^ single assessment numerical evaluation (SANE),^43^ and age-adjusted Constant score^9^ were recorded at 6 and 12 months. Radiologic union, which was defined as complete disappearance of the fracture line, and acromioclavicular joint changes were recorded.

## Results

### Patient demographics

Patient demographics are summarized in Table I. There were 15 patients (9 Males and 6 Females), with a mean age of 44.7 years [range, 21 to 62 years]. All patients are right-handed. All patients were followed-up to a minimum of 12 months. There were 12 Neer type II and 3 Neer type V fractures. The identified mechanisms were sport-related in 12 cases, and a fall from standing height in the other 3 cases.

**Table I:** Summary of patient demographic factors, including age, sex, affected side, trauma mechanism, and fracture type.

Patient	Age	Sex	Affected side	Mechanism	Fracture type (Neer)
1	37	Male	Right	Skiing accident	5
2	29	Male	Left	Skiing accident	2
3	25	Female	Right	Combat sport	2
4	41	Male	Left	Moutainbike fall	2
5	51	Male	Left	Surfing	2
6	53	Male	Left	Bicycle fall	5
7	53	Female	Right	Fall from height	2
8	61	Male	Right	Fall from height	2
9	57	Male	Left	Mountainbike fall	2
10	21	Male	Right	Football injury	2
11	25	Male	Right	Skateboard injury	2
12	62	Female	Left	Bicycle fall	2
13	51	Female	Left	Fall from height	5
14	47	Female	Left	Motorbike accident	2
15	58	Female	Left	Mountainbike fall	2

### Clinical assessment

By 6 months, all patients recovered full range of motion apart from one patient (patient 7) who developed transient stiffness that fully resolved at the final 12 months follow-up. No complications or reoperations were noted.

Mean VAS was 0 at 6 months, mean SANE score was 92.7 [± 10.7, range 60 to 100] at 6 months, and 98.2 [± 5.2, range 80 to 100] at 12 months. The adjusted Constant score was 99.0 [± 1.9, range 94 to 100] at 12 months (Table II).

**Table II:** Summary of postoperative results at 6 and 12 months, including clinical scores and radiologic workup.

	SANE score 6 months	SANE score 12 months	Constant score 12 months	Time to union	AC joint changes
1	100	100	97	3	No
2	95	100	94	6	No
3	100	100	97	3	No
4	95	99	100	3	No
5	100	95	100	3	No
6	99	99	100	12	No
7	60	80	100	12	No
8	90	100	100	12	No
9	90	100	100	6	No
10	98	100	100	6	No
11	100	100	100	3	No
12	80	100	100	12	No
13	90	100	100	6	No
14	95	100	100	3	No
15	99	100	97	3	No
Mean	92.7	98.2	99.0	6.2	

*SANE*, Single Assessment numerical evaluation; *AC*, acromio-clavicular.

### Radiographic assessment

**Figure 5:**
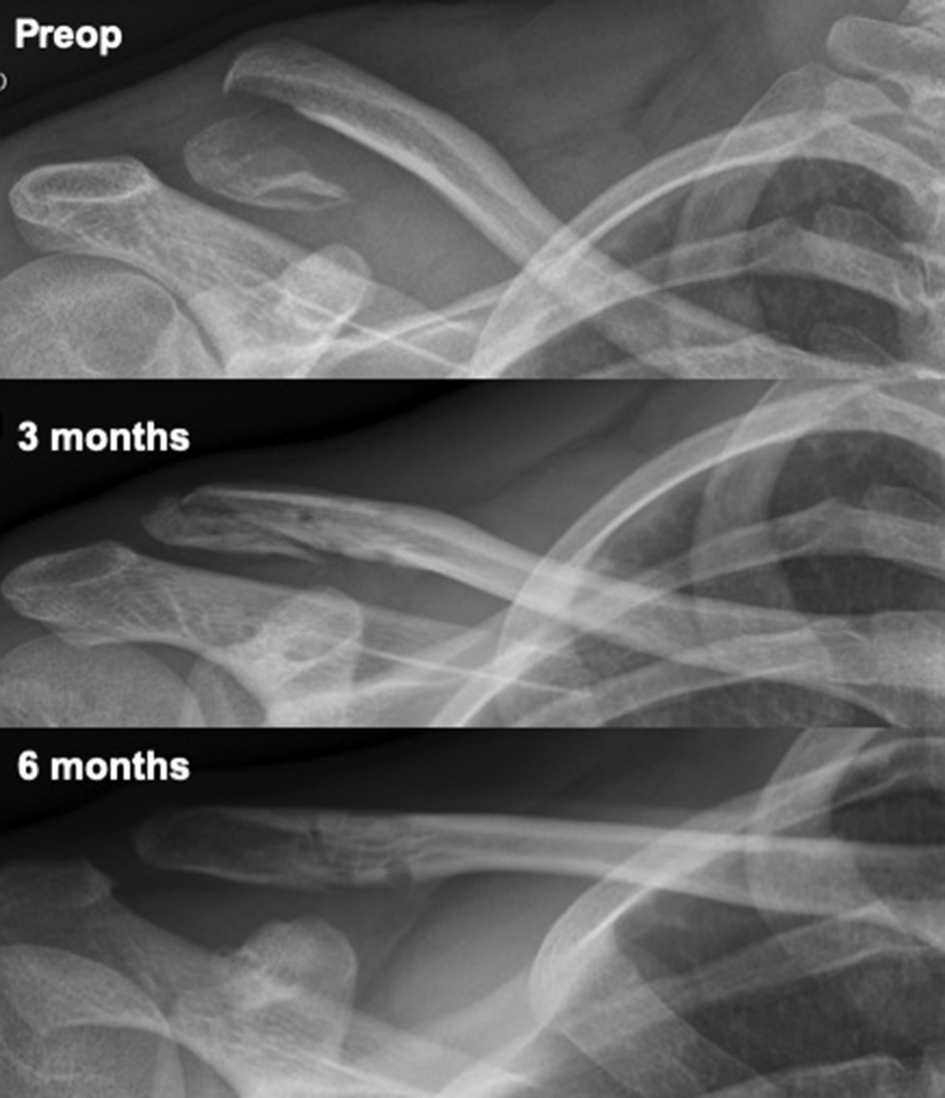
Radiographs showing a displaced Neer type II distal clavicle fracture, preoperatively and postoperatively at 3 and 6 months. Full union was achieved between 3 and 6 months.

Fractures consolidated in 93% of the cases, with union happening between the 3 and 6 months follow-up [range 3 to 12 months] (Fig. 5). One patient (patient 6) developed an asymptomatic malunion, related to early sling discontinuation and returned to rowing exercises after only 3 weeks. Correlation analysis between radiologic union and functional scores showed moderate negative correlation between time to union with 6 months SANE score (r = –0.7, *P* = .003) but none with 1 year SANE or adjusted Constant scores (r = –0.13, *P* = .6 and r = 0.35, *P* = .2, respectively). No degenerative changes were noted in the acromioclavicular joint, although follow-up time may have been short to fully assess this criterion.

## Discussion

We describe an all-suture technique to treat displaced distal clavicle fractures with satisfactory 1-year results. It achieves good clinical outcomes with a high union rate, and most of all, no implant-related complications or reoperations for implant removal as with other techniques.

Locking plates are widely used with good reported outcomes.^12^^,^^15^^,^^18^^,^^20^^,^^22^^,^^27^^,^^42^^,^^46^^,^^52^ However, these plates may produce discomfort or pain and often require removal.^37^ Moreover, bone purchase with screws can be hard to achieve in the small or comminuted distal fragment. Hook plates may avoid this problem and have also shown overall satisfactory results in several studies.^1^^,^^23^^,^^40^ However, complications are frequent, including subacromial shoulder impingement and rotator cuff lesion, stress shielding, and osteolysis.^28^^,^^29^^,^^51^ One study reported a complication rate of 63%, mainly due to implant irritation.^37^ Therefore, in most cases, plate removal is necessary. Two studies compared plates and osteosuture techniques and showed similar outcomes with a lower complication rate in the latter.^7^^,^^19^ Pinning fixation has been reported but is associated with a high complication rate such as k-wire irritation and migration^37^^,^^44^ and is therefore not recommended.

Cortical button fixation devices have also been reported to lead to good results.^4^^,^^8^^,^^10^^,^^21^^,^^30^^,^^39^^,^^49^^,^^53^ Although these devices show a lower rate of complication and superior biomechanical stability compared to locking plate fixation,^16^^,^^36^^,^^45^^,^^47^ the coracoid fracture is a unique complication to this technique related to coracoid drilling.^16^^,^^21^^,^^31^^,^^36^^,^^45^^,^^47^ Other complications have been reported, such as loss of reduction and failure of coracoid button fixation.^31^ Moreover, cortical button devices only allow control of vertical stability, but not horizontal and rotational stability, unless a graft is added to strengthen the construct.^48^

Several all-suture techniques have been described. Coracoclavicular loop alone has been used in some studies.^7^^,^^17^^,^^19^^,^^37^^,^^50^ However, as with cortical button fixation, they do not control horizontal and rotational stability. Three all-suture techniques have been described that stabilize the coracoclavicular ligaments and the fracture site. Soliman reported in 2013 a technique with 3 Ethibond sutures under the coracoid and around the clavicle (UCAC) loop.^38^ Duralde, in 2014, reported another suture technique using 2 FiberWire sutures under the coracoid and 1 or 2 FiberWire across the fracture fragments in a figure-of-8.^13^ Sarda, in 2019, reported a modified UCAC technique with one loop around the coracoid and the clavicle and another loop around the coracoid and through the clavicle.^34^ Although these 3 previous techniques stabilize the fracture vertically and horizontally, some of them use either a single coracoclavicular strand or do not stabilize the fracture in multiple planes.

Although there are no direct comparisons between the different published similar techniques, the presented one provides multiple theoretical advantages. First, the frame-type coracoclavicular cerclage and vertical clavicle drilling using 4 no6 Ethibond strands offer a robust vertical and axial rotational stability, closely restoring anatomical biomechanics of the coracoclavicular ligaments, and has been proved to be biomechanically superior to a cortical button device in the acromioclavicular joint (ACJ) dislocation stabilization.^25^ Second, fixing the fracture site with a figure-of-8 and a figure-of-0 technique allows horizontal and rotational stability.^14^ Finally, this technique avoids implant removal or implant-related complications, as stated above. The choice of type of sutures was made according to recent biomechanical findings^41^; A flat-braided suture such as SutureTape offers more rigidity to the fracture site with less creep, while Ethibond offers more elasticity, replicating ligament properties more closely.

This study presented some limitations. First, the aim of this pilot study was to describe a new technique. Although it is a prospective series, the number of cases remains limited. Second, and for the same reason, there is no control group treated with a plate or cortical button, which would have been useful to confirm some of the theoretical advantages of this technique. However, a control group is not needed to prove the main advantages of this all-suture technique since it avoids specific complications unique to other routine fixation techniques (such as a coracoid fracture from drilling or reoperations for plate removal). Nevertheless, further larger comparative series are needed to extend these promising results.

## Conclusion

Excellent clinical and radiological outcomes can be achieved with this minimally invasive all-suture fixation technique for displaced distal clavicle fractures, which allows for an anatomic reduction and stable fixation. This pilot study showed low complications and a high level of union after a follow-up of 1 year. Among the numerous advantages are a smaller exposure than for plate fixation, avoidance of hardware-related complications such as screw failure, coracoid fracture from drilling, or rotator cuff damage caused by hook-plates. Furthermore, it avoids a reoperation to remove symptomatic hardware.

## Disclaimers:

Funding: No funding was disclosed by the authors.

Conflicts of interest: The authors, their immediate families, and any research foundation with which they are affiliated have not received any financial payments or other benefits from any commercial entity related to the subject of this article.

## References

[1] Asadollahi S., Bucknill A. (2019). Hook Plate Fixation for Acute Unstable Distal Clavicle Fracture: A Systematic Review and Meta-analysis. J Orthop Trauma.

[2] Banerjee R., Waterman B., Padalecki J., Robertson W. (2011). Management of distal clavicle fractures. J Am Acad Orthop Surg.

[3] Bishop J.Y., Jones G.L., Lewis B., Pedroza A., MOON Shoulder Group (2015). Intra- and interobserver agreement in the classification and treatment of distal third clavicle fractures. Am J Sports Med.

[4] Blake M.H., Lu M.T., Shulman B.S., Glaser D.L., Huffman G.R. (2017). Arthroscopic Cortical Button Stabilization of Isolated Acute Neer Type II Fractures of the Distal Clavicle. Orthopedics.

[5] Boehm T.D., Kirschner S., Fischer A., Gohlke F. (2003). The relation of the coracoclavicular ligament insertion to the acromioclavicular joint: a cadaver study of relevance to lateral clavicle resection. Acta Orthop Scand.

[6] Boonard M., Sumanont S., Arirachakaran A., Sikarinkul E., Ratanapongpean P., Kanchanatawan W., Kongtharvonskul J. (2018). Fixation method for treatment of unstable distal clavicle fracture: systematic review and network meta-analysis. Eur J Orthop Surg Traumatol.

[7] Chen C.Y., Yang S.W., Lin K.Y., Lin K.C., Tarng Y.W., Renn J.H., Lai C.H. (2014). Comparison of single coracoclavicular suture fixation and hook plate for the treatment of acute unstable distal clavicle fractures. J Orthop Surg Res.

[8] Cisneros L.N., Reiriz J.S. (2017). Management of unstable distal third clavicle fractures: clinical and radiological outcomes of the arthroscopy-assisted conoid ligament reconstruction and fracture cerclage with sutures. Eur J Orthop Surg Traumatol.

[9] Constant C.R., Murley A.H. (1987). A clinical method of functional assessment of the shoulder. Clin Orthop Relat Res.

[10] Dedeoğlu S.S., İmren Y., Çabuk H., Çakar M., Arslan S.M., Esenyel C.Z. (2017). Results of minimal invasive coracoclavicular fixation by double button lift-up system in Neer type II distal clavicle fractures. J Orthop Surg (Hong Kong).

[11] Dixon J.S., Bird H.A. (1981). Reproducibility along a 10 cm vertical visual analogue scale. Ann Rheum Dis.

[12] Dong W.W., Zhao X., Mao H.J., Yao L.W. (2019). [Minimally-invasive internal fixation for mid-lateral 1/3 clavicle fracture with distal clavicular anatomic locking plate]. Zhongguo Gu Shang.

[13] Duralde X.A., Pennington S.D., Murray D.H. (2014). Interfragmentary suture fixation for displaced acute type II distal clavicle fractures. J Orthop Trauma.

[14] Dyrna F., Imhoff F.B., Haller B., Braun S., Obopilwe E., Apostolakos J.M., Morikawa D., Imhoff A.B., Mazzocca A.D., Beitzel K. (2018). Primary Stability of an Acromioclavicular Joint Repair Is Affected by the Type of Additional Reconstruction of the Acromioclavicular Capsule. Am J Sports Med.

[15] Fan J., Zhang Y., Huang Q., Jiang X., He L. (2017). Comparison of Treatment of Acute Unstable Distal Clavicle Fractures Using Anatomical Locking Plates with Versus without Additional Suture Anchor Fixation. Med Sci Monit.

[16] Flinkkilä T., Heikkilä A., Sirniö K., Pakarinen H. (2015). TightRope versus clavicular hook plate fixation for unstable distal clavicular fractures. Eur J Orthop Surg Traumatol.

[17] Friedman D.J., Barron O.A., Catalano L., Donahue J.P., Zambetti G. (2008). Coracoclavicular stabilization using a suture anchor technique. Am J Orthop (Belle Mead NJ).

[18] Govindasamy R., Kasirajan S., Doke T. (2017). Functional Results of Unstable (Type 2) Distal Clavicle Fractures Treated with Superior Anterior Locking Plate. Arch Bone Jt Surg.

[19] Hsu K.H., Tzeng Y.H., Chang M.C., Chiang C.C. (2018). Comparing the coracoclavicular loop technique with a hook plate for the treatment of distal clavicle fractures. J Shoulder Elbow Surg.

[20] Ibrahim S., Meleppuram J.J. (2017). Retrospective study of superior anterior plate as a treatment for unstable (Neer type 2) distal clavicle fractures. Rev Bras Ortop.

[21] Kapicioglu M., Erden T., Bilgin E., Bilsel K. (2021). All arthroscopic coracoclavicular button fixation is efficient for Neer type II distal clavicle fractures. Knee Surg Sports Traumatol Arthrosc.

[22] Kapil-Mani K.C., Acharya P., Arun S. (2018). Precontoured Clavicular Locking Plate with Broad Lateral End: A Newly Designed Plate for Lateral Third Clavicle Fractures. Malays Orthop J.

[23] Kirsch J.M., Blum L., Hake M.E. (2018). Distal Clavicle Fractures: Open Reduction and Internal Fixation With a Hook Plate. J Orthop Trauma.

[24] Lädermann A., Grosclaude M., Lübbeke A., Christofilopoulos P., Stern R., Rod T., Hoffmeyer P. (2011). Acromioclavicular and coracoclavicular cerclage reconstruction for acute acromioclavicular joint dislocations. J Shoulder Elbow Surg.

[25] Lädermann A., Gueorguiev B., Stimec B., Fasel J., Rothstock S., Hoffmeyer P. (2013). Acromioclavicular joint reconstruction: a comparative biomechanical study of three techniques. J Shoulder Elbow Surg.

[26] Laux C.J., Villefort C., El Nashar R., Farei-Campagna J.M., Grubhofer F., Bouaicha S., Gerber C., Meyer D.C., Wieser K. (2021). Stand-alone coracoclavicular suture repair achieves very good results in unstable distal clavicle fractures at a minimum follow-up of 1 year. J Shoulder Elbow Surg.

[27] Lee S.K., Lee J.W., Song D.G., Choy W.S. (2013). Precontoured locking plate fixation for displaced lateral clavicle fractures. Orthopedics.

[28] Lin H.Y., Wong P.K., Ho W.P., Chuang T.Y., Liao Y.S., Wong C.C. (2014). Clavicular hook plate may induce subacromial shoulder impingement and rotator cuff lesion--dynamic sonographic evaluation. J Orthop Surg Res.

[29] Lopiz Y., Checa P., García-Fernández C., Valle J., Vega M.L., Marco F. (2019). Complications with the clavicle hook plate after fixation of Neer type II clavicle fractures. Int Orthop.

[30] Loriaut P., Moreau P.E., Dallaudière B., Pélissier A., Vu H.D., Massin P., Boyer P. (2015). Outcome of arthroscopic treatment for displaced lateral clavicle fractures using a double button device. Knee Surg Sports Traumatol Arthrosc.

[31] Milewski M.D., Tompkins M., Giugale J.M., Carson E.W., Miller M.D., Diduch D.R. (2012). Complications related to anatomic reconstruction of the coracoclavicular ligaments. Am J Sports Med.

[32] Neer C.S. (1968). Fractures of the distal third of the clavicle. Clin Orthop Relat Res.

[33] Ockert B., Wiedemann E., Haasters F. (2015). Laterale Klavikulafraktur. Klassifikationen und Therapieoptionen [Distal clavicle fractures. Classifications and management]. Unfallchirurg.

[34] Sarda P. (2019). Lateral Clavicle Fractures with Coracoclavicular Ligament Disruption (Neer's Type IIB): Review of Literature and a New Technique for All-Suture Fixation. Indian J Orthop.

[35] Seppel G., Lenich A., Imhoff A.B. (2014). Die laterale Klavikulafraktur [Distal clavicle fracture]. Oper Orthop Traumatol.

[36] Turkmen M. (2015). Anatomic locking plate and coracoclavicular stabilization with suture endo-button technique is superior in the treatment of Neer Type II distal clavicle fractures. Eur J Orthop Surg Traumatol.

[37] Singh A., Schultzel M., Fleming J.F., Navarro R.A. (2019). Complications after surgical treatment of distal clavicle fractures. Orthop Traumatol Surg Res.

[38] Soliman O., Koptan W., Zarad A. (2013). Under-coracoid-around-clavicle (UCAC) loop in type II distal clavicle fractures. Bone Joint J.

[39] Struhl S., Wolfson T.S. (2016). Closed-Loop Double Endobutton Technique for Repair of Unstable Distal Clavicle Fractures. Orthop J Sports Med.

[40] Şükür E., Öztürkmen Y., Akman Y.E., Güngör M. (2016). Clinical and radiological results on the fixation of Neer type 2 distal clavicle fractures with a hook plate. Acta Orthop Traumatol Turc.

[41] Taha M.E., Schneider K., Clarke E.C., O'Briain D.E., Smith M.M., Cunningham G., Cass B., Young A.A. (2020). A Biomechanical Comparison of Different Suture Materials Used for Arthroscopic Shoulder Procedures. Arthroscopy.

[42] Vaishya R., Vijay V., Khanna V. (2017). Outcome of distal end clavicle fractures treated with locking plates. Chin J Traumatol.

[43] Williams G.N., Gangel T.J., Arciero R.A., Uhorchak J.M., Taylor D.C. (1999). Comparison of the Single Assessment Numeric Evaluation method and two shoulder rating scales. Outcomes measures after shoulder surgery. Am J Sports Med.

[44] Wu C.C. (2012). Tension band wiring versus Knowles pinning for non-union of type-2 distal clavicle fractures. J Orthop Surg (Hong Kong).

[45] Xiong J., Chen J.H., Dang Y., Zhang D.Y., Fu Z.G., Zhang P.X. (2018). Treatment of unstable distal clavicle fractures (Neer type II): A comparison of three internal fixation methods. J Int Med Res.

[46] Xu H., Chen W.J., Zhi X.C., Chen S.C. (2019). Comparison of the efficacy of a distal clavicular locking plate with and without a suture anchor in the treatment of Neer IIb distal clavicle fractures. BMC Musculoskelet Disord.

[47] Yagnik G.P., Brady P.C., Zimmerman J.P., Jordan C.J., Porter D.A. (2019). A biomechanical comparison of new techniques for distal clavicular fracture repair versus locked plating. J Shoulder Elbow Surg.

[48] Yagnik G.P., Jordan C.J., Narvel R.R., Hassan R.J., Porter D.A. (2019). Distal Clavicle Fracture Repair: Clinical Outcomes of a Surgical Technique Utilizing a Combination of Cortical Button Fixation and Coracoclavicular Ligament Reconstruction. Orthop J Sports Med.

[49] Yagnik G.P., Porter D.A., Jordan C.J. (2018). Distal Clavicle Fracture Repair Using Cortical Button Fixation With Coracoclavicular Ligament Reconstruction. Arthrosc Tech.

[50] Yang S.W., Lin L.C., Chang S.J., Kuo S.M., Hwang L.C. (2011). Treatment of acute unstable distal clavicle fractures with single coracoclavicular suture fixation. Orthopedics.

[51] Zhang L., Xiao H., Gao Y., Zhang H., Zhang L., Tang P. (2018). Late function and complications of hook plate implantation for distal-third clavicle fractures : a retrospective study. Acta Orthop Belg.

[52] Zhang Y.F., Mi M., Zhang J., Guo Q., Gong M.Q., Huang Q., Jiang X.Y. (2019). [Case-control study on single locking plate and locking plate with suture anchors for the treatment of unstable distal clavicle fractures]. Zhongguo Gu Shang.

[53] Zheng Y.R., Lu Y.C., Liu C.T. (2019). Treatment of unstable distal-third clavicule fractures using minimal invasive closed-loop double endobutton technique. J Orthop Surg Res.

